# Attitudes about COVID-19 Lockdown among General Population, France, March 2020

**DOI:** 10.3201/eid2701.201377

**Published:** 2021-01

**Authors:** Patrick Peretti-Watel, Valérie Seror, Sébastien Cortaredona, Odile Launay, Jocelyn Raude, Pierre Verger, François Beck, Stéphane Legleye, Olivier L’Haridon, Jeremy Ward, for the Coronavirus and Confinement, Enquête Longitudinale, COCONEL, Study Group

**Affiliations:** VITROME (Vecteurs–Infections Tropicales et Méditerranéennes), Institut Méditerranée Infection, Aix Marseille Université, Marseille, France (P. Peretti-Watel, V. Seror, S. Cortaredona, J. Ward);; Observatoire Régional de la Santé Provence-Alpes-Côte d'Azur, Marseille (P. Peretti-Watel, P. Verger);; Centre d’Investigation Clinique Cochin-Pasteur, Paris, France (O. Launay) ;; École des Hautes Études en Santé Publique, Rennes (J. Raude);; Centre de Recherche en Épidémiologie et Santé des Populations, Villejuif, France (F. Beck, S. Legleye);; Université de Rennes, Rennes (O. L’Haridon);; Groupe d’Étude des Méthodes de l’Analyse Sociologique de la Sorbonne, Paris (J. Ward)

**Keywords:** COVID-19, coronavirus disease, SARS-CoV-2, severe acute respiratory syndrome coronavirus 2, viruses, respiratory infections, zoonoses, lockdown, France, social inequalities

## Abstract

Because the effectiveness of a coronavirus disease lockdown in curbing coronavirus disease spread depends on public support, acquiring real-time information about the way populations reacted to the lockdown is crucial. In France, such public support remained fragile among low-income persons, probably because the lockdown exacerbated preexisting social inequalities and conflicts.

During the spring of 2020, because of the coronavirus disease (COVID-19) pandemic, >3 billion persons worldwide lived under lockdown, and many of them were probably angry, uncertain, and distrustful of their national leaders (*1*). Thus, acquiring real-time information about the way populations react and comply to such stringent measures across different socio-economic groups and sociocultural contexts is crucial (*2*). Social acceptability is especially important in the case of France, because the general population did not adhere to governmental recommendations against the previous 2009 influenza A(H1N1) pandemic, during which only 8% of adults complied with the mass vaccination campaign promoted by health authorities (*3*,*4*).

To investigate attitudes toward the lockdown among the general population in France, we conducted a cross-sectional online survey among a nationally representative sample (N = 1,012) of residents >18 years of age (Table). The survey was administered during March 27–29, about 10 days after the nationwide lockdown was introduced. To limit selection bias, categories of persons who are less prone to participate in internet surveys (e.g., workers and older persons) were oversampled, and the invitational email did not mention the theme of the survey. In terms of response bias, self-administered questionnaires tend to yield fewer reports in the socially desirable direction than do interviewer-administered questionnaires, and online surveys might have the lowest levels of social-desirability bias (*5*). We computed participants’ equivalized household income per month, taking into account household size and composition. Low income refers to the bottom quartile, medium income to the second and third quartiles, and high income to the top quartile. Participants were asked to express their level of agreement toward 12 statements related to lockdown. We asked them whether they were experiencing financial difficulties because of the lockdown. We also asked for household size and housing surface area to identify participants confined in an overcrowded household.

**Table:** Opinion about the coronavirus disease lockdown among 1,012 respondents to the Coronavirus and Confinement: Enquête Longitudinale (COCONEL) survey, France, March 27–29, 2020*

Statement or condition	% Respondents who agreed with statement, by income level (+ MoE)				p value
	Overall	Lower income, n = 216	Medium income, n = 566	Higher income, n = 230	
The lockdown:					
Is the only effective way to fight the epidemic	88 (+2)	81 (+5)	90 (+2)	93 (+3)	<0.001
Should last several more weeks to be effective	93 (+1)	89 (+4)	94 (+2)	98 (+2)	<0.01
Should be strengthened to be effective	80 (+2)	75 (+6)	82 (+3)	81 (+5)	NS
Is disproportionate considering the real gravity of the epidemic	20 (+2)	35 (+6)	18 (+3)	10 (+4)	<0.001
Should be less coercive to be more acceptable	22 (+3)	33 (+6)	21 (+3)	13 (+4)	<0.001
Is the consequence of the lack of hospital resources	66 (+3)	72 (+6)	68 (+4)	54 (+6)	<0.001
Could have been avoided by the widespread wearing of masks	50 (+3)	61 (+7)	51 (+4)	40 (+6)	<0.001
Could be replaced by mass screening tests	65 (+3)	74 (+6)	65 (+4)	60 (+6)	<0.01
Has already disastrous economic consequences	93 (+2)	93 (+3)	91 (+2)	96 (+2)	NS
Will cause family tragedies	76 (+3)	78 (+6)	76 (+4)	75 (+6)	NS
Causes too much restriction on civil liberties	41 (+3)	58 (+7)	40 (+4)	28 (+6)	<0.001
Is an opportunity to develop local solidarity	91 (+2)	92 (+4)	90 (+2)	91 (+4)	NS
Experiencing financial difficulties because of the lockdown	19 (+2)	40 (+7)	16 (+3)	6 (+3)	<0.001
Confined in an overcrowded household†	9 (+2)	23 (+6)	7 (+2)	1 (+1)	<0.001

*Sample was randomly drawn from online research panel of >750,000 nationally representative households of the general population in France, developed and maintained by the Institut Français d’Opinion Publique, a survey research firm (https://www.ifop.com). Collected data were weighted to match official national census statistics for sex, age, occupation, size of population in the area of residence, and region. The study design was approved by the Ethics Committee of the University Hospital Institute Méditerranée Infection (approval no. 2020-018). MoE, margin of error at 95% confidence level; NS, not statistically significant.  †Defined as <194 square feet per capita.

Most participants supported the current lockdown as the only effective way to fight the epidemic and the need to maintain it for several more weeks; however, this support was significantly lower (p<0.001 by χ^2^ test) among low-income respondents (Table). Strong support was observed across all income groups in favor of strengthening controls to making the lockdown more effective. Only a few respondents (more frequently low-income respondents) expressed open criticisms, including statements indicating that the lockdown is “disproportionate considering the real gravity of the epidemic” (35% among low-income respondents vs. 10% among high-income respondents) and that it should be less coercive to be more acceptable (33% among low-income respondents vs. 13% among high-income respondents).

However, the consensus for the lockdown was based on the fact that it appeared a stopgap measure implemented because of a lack of alternatives: 66% of respondents agreed that the lockdown was the consequence of the lack of hospital resources, 65% agreed that mass testing could replace the lockdown, and 50% considered that the lockdown could have been “avoided by the widespread wearing of masks.” Once again, such statements were more frequent among low-income respondents. Similarly, although all socioeconomic groups acknowledged some major drawbacks (including disastrous economic consequences and family tragedies), low-income respondents were more likely than high-income respondents to state that the lockdown was causing “too much restriction on civil liberties” (58% vs. 28%). 

Social differences in attitudes toward the lockdown are probably related to practical differences in persons’ living conditions during the lockdown. After 10 days of confinement, 40% of respondents in the low-income group were already reporting financial difficulties because of the lockdown (compared with 6% among high-income respondents). In terms of housing conditions, 9% of participants were confined in an overcrowded household, but that was the case for 23% of low-income respondents (compared with 1% of high-income respondents). Overcrowded housing can impair mental health, and the lockdown made crowded situations even more unbearable because engaging in outdoor activities typically is the easiest way to cope with such situations (*6*,*7*).

In France, as in most other countries, the COVID-19 pandemic fueled contradictory information and intense controversies in traditional and social media. Our survey suggests that a social consensus has been maintained in France in favor of the national lockdown and that excessive politicization of public health has been avoided so far (*8*). However, this consensus remained fragile. First, opinion might have changed if the public got the impression that authorities did not promote alternatives fast enough to end the confinement period. Second, as exemplified by the lower support observed in the poorest groups, the pandemic and the lockdown both exacerbated existing social inequalities and conflicts; besides social inequalities in terms of income and housing conditions, hospital workers in France had been on strike for months during the previous year demanding more resources, and many opponents accused the government of impinging on civil liberties during the so-called “yellow vests” protest movement.

In summary, in late March, most persons in France did support the lockdown; however, such consensus remained fragile because of existing social inequalities and conflicts. Continuous monitoring of population’s attitudes and practices during the pandemic will remain key for guiding the public health response (*9*) and communication strategy (*10*).
